# Seqpac: a framework for sRNA-seq analysis in R using sequence-based counts

**DOI:** 10.1093/bioinformatics/btad144

**Published:** 2023-03-21

**Authors:** Signe Skog, Lovisa Örkenby, Unn Kugelberg, Anita Öst, Daniel Nätt

**Affiliations:** Division of Cell Biology, Department of Biomedical and Clinical Sciences, Linkoping University, Linkoping SE-58185, Sweden; Division of Cell Biology, Department of Biomedical and Clinical Sciences, Linkoping University, Linkoping SE-58185, Sweden; Division of Cell Biology, Department of Biomedical and Clinical Sciences, Linkoping University, Linkoping SE-58185, Sweden; Division of Cell Biology, Department of Biomedical and Clinical Sciences, Linkoping University, Linkoping SE-58185, Sweden; Division of Cell Biology, Department of Biomedical and Clinical Sciences, Linkoping University, Linkoping SE-58185, Sweden

## Abstract

**Motivation:**

Feature-based counting is commonly used in RNA-sequencing (RNA-seq) analyses. Here, sequences must align to target features (like genes or non-coding RNAs) and related sequences with different compositions are counted into the same feature. Consequently, sequence integrity is lost, making results less traceable against raw data.

Small RNA (sRNA) often maps to multiple features and shows an incredible diversity in form and function. Therefore, applying feature-based strategies may increase the risk of misinterpretation. We present a strategy for sRNA-seq analysis that preserves the integrity of the raw sequence making the data lineage fully traceable. We have consolidated this strategy into Seqpac: An R package that makes a complete sRNA analysis available on multiple platforms. Using published biological data, we show that Seqpac reveals hidden bias and adds new insights to studies that were previously analyzed using feature-based counting.

We have identified limitations in the concurrent analysis of RNA-seq data. We call it the traceability dilemma in alignment-based sequencing strategies. By building a flexible framework that preserves the integrity of the read sequence throughout the analysis, we demonstrate better interpretability in sRNA-seq experiments, which are particularly vulnerable to this problem. Applying similar strategies to other transcriptomic workflows may aid in resolving the replication crisis experienced by many fields that depend on transcriptome analyses.

**Availability and implementation:**

Seqpac is available on Bioconductor (https://bioconductor.org/packages/seqpac) and GitHub (https://github.com/danis102/seqpac).

## 1 Introduction

Many biological and medical fields suffer from low reproducibility, commonly called the replication crisis (Loken and Gelman 2017). Concerns are now being raised in methodological fields, such as bioinformatics (Boulesteix et al. 2020; Buchka et al. 2021). Transcriptome-scale RNA-sequencing (RNA-seq) has quickly become the golden standard for studying changes in genome expression. Even though data traceability, or provenance, has long been recognized as a scientific challenge (Buneman et al. 2000) little has been done to develop RNA-seq workflows with clean data lineages, where results are easily verifiable against raw data. Developing strategies to enhance back-traceability may therefore aid in solving the current replication crisis.

**Figure 1:**
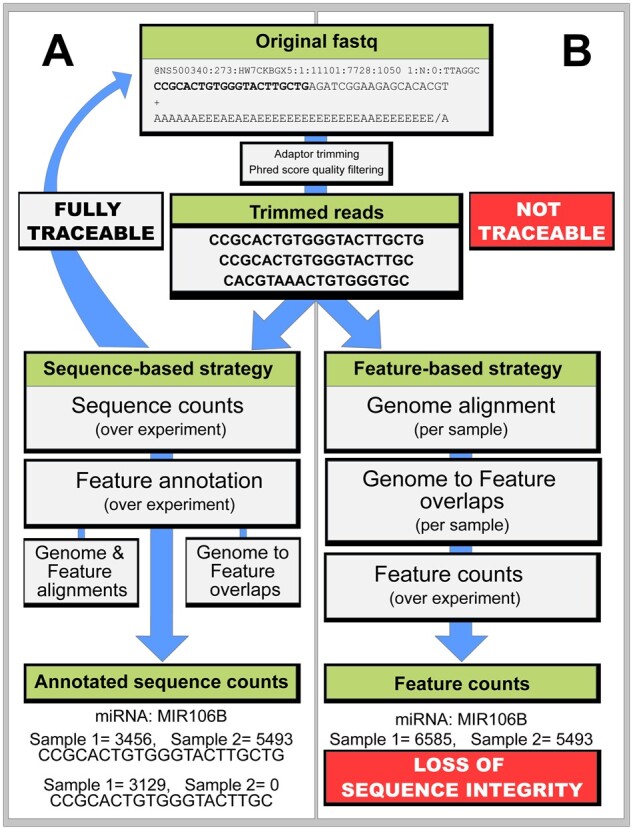
Differences between sequence-based and feature-based counting strategies. In sequence-based strategies (A), a count table of unique sequences across all samples is created. Genome and feature alignments are applied to annotate and classify these sequences. Sequence integrity is preserved and results can be further annotated or validated against raw data, at any point. In feature-based strategies (B), which are more common, read sequences are first aligned to a species genome. Counts are based on the overlaps between genome coordinates of the reads and known features of a reference genome (e.g. genes or miRNA). When features multimap, users must prioritize one feature class over another, or randomly assign a coordinate. Sequences that fail to align are discarded. As related, but different, sequences are counted into the same feature, sequence integrity is lost. Thus, the results are challenging to trace against raw data and cannot be annotated further. Seqpac is designed to preserve the sequence integrity in sRNA-seq data analysis by applying a sequence-based counting strategy (A)

The first step in most RNA-seq workflows, after the removal of adaptor sequences (trimming), is to align read sequences against the genome or transcriptome of a target species, allowing for a user-defined number of mismatches and sequence lengths (Fig. 1) (Conesa et al. 2016). Sequences that fail to align are commonly disregarded and sequences of differing lengths, with or without mismatches, are pooled into features, such as genes, exons, or small RNAs (sRNAs). Analyses commonly focus on one well-defined feature class, such as genes or miRNA, thereby, prioritizing this class over others. Importantly, performing the analysis on feature counts, where a count constitutes of related but not identical sequences, may cloud the interpretation. The final result may indicate that there is a group difference in a particular gene but will not reveal differential mismatches (e.g. genetic variants) or read lengths (e.g. degradation) between groups (Fig. 1). Sequences that failed to align, but may have influenced the result (e.g. contamination), are neglected.

Traceability against raw data is seldom requested at publication and may even be practically impossible for independent investigators, such as a reviewer. This is not because it is impossible in theory. Well-designed tools provide output options that, when carefully configured, may be used to create intact data lineages. In practice, however, such workflows would involve multiple modules (Conesa et al. 2016), resulting in unnecessarily complicated data lineages. Together with feature counting (Fig. 1), this is a probable reason why complete data lineages are not requested at the publication of RNA-seq analyses. We call this the traceability dilemma in alignment-based sequencing analysis.

Raw data in sequencing experiments are commonly stored in the fastq format. Building workflows with intact data lineages, would in practice mean that results are easily validated against corresponding fastq file. One simple solution to obtain that is to count sequences rather than features, and to avoid aligning sequences early in the analysis, only to apply such mapping as means to interpret changes in raw sequences (Fig. 1).

Here, we present Seqpac, a novel framework for sequence analysis intended to preserve the integrity of raw data by using sequence counts. Seqpac is implemented as a package in R, from adapter trimming to the visualization of group differences. This makes it accessible on multiple platforms, including Windows, Mac, and Linux.

## 2 Implementation

**Figure 2:**
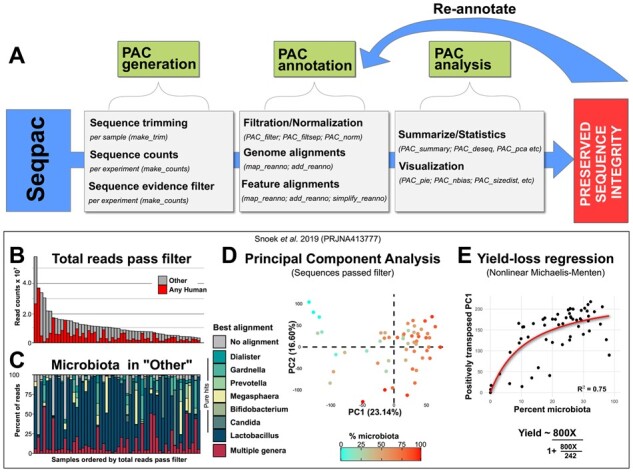
Seqpac in action. (A) A typical Seqpac workflow. Seqpac is designed to preserve sequence integrity by avoiding a feature-based alignment strategy that normally disregards sequences that fail to align to a target genome (e.g. human). Among other things, this means that results are easily re-annotated. More details on how to build workflows are available in our vignette guide that accompanies the package. (B–E) To demonstrate how Seqpac may impact how data are interpreted, we downloaded the (Snoek et al. 2019) dataset that studied clinical self-samples taken by women with or without cervical cancer. (B) In this study, a feature-based strategy was used. The authors did not report additional species in the samples that could have affected the result and their interpretations. Samples varied greatly in the total number of reads and in the proportion of reads aligning to the human genome. Since Seqpac saves all sequences, not only those that align to the human genome, Basic Local Alignment Search Tool (BLAST) at NCBI (Sayers et al. 2022) were used to obtain more information about nonaligned sequences. Many sequences originated from the vaginal microbiota. (C) Results from multi-genome alignment using Seqpac’s reannotation workflow showed that non-aligning sequences indeed aligned with species commonly found in the vaginal microbiota. (D) PCA showed that the microbiota explained much of the variance in the study. (E) The relationship between the main component (PC1) and the proportion microbiota closely followed a Yield-Loss regression curve, commonly used to model the effect of weed density on crop yield (Ritz et al. 2015). This demonstrates the importance of preserving sequence integrity to account for nonaligning sequences, for example by statistical modeling. More examples of the benefits of sequence-based versus feature-based strategies are given in Supplementary Figs S3 and S5. All code for reproducing the analysis is available in Supplementary File S3

Currently, Seqpac is optimized for the analysis of sRNA-seq, using fastq as input (Fig. 1) but can be adapted for other types of sequencing experiments. It builds on a unique set of functions (Supplementary Table S1) for constructing and analyzing a PAC-object, containing the experimental metadata (Pheno), Annotation-, and Counts table (Supplementary Fig. A1). This workflow comes with adaptor trimming, outperforming conventional alternatives (Supplementary Fig. S2). In the typical workflow (Fig. 2A), Seqpac implements its framework of objects and functions to preserve the original fastq sequence. In practice, this means that results can easily be re-analyzed any number of times, for example by using Basic Local Alignment Search Tool (BLAST) (Sayers et al. 2022) or other metatranscriptomic tools to obtain more information on unaligned sequences. Seqpac provides the re-annotation workflow that is specifically designed for sequences in the PAC-object (Supplementary Fig. S1C). Among other unique capabilities, which are demonstrated in the examples (Fig. 2B–E, Supplementary Figs S3 and S5), Seqpac contains an innovative approach for focusing and dividing data by sample group or sequence classes (Supplementary Fig S1B).

Many workflows for sRNA-seq analysis have inherited elements from regular RNA-seq analysis, such as feature-based counting (Fig. 1), even though the traceability dilemma is more prevalent for several reasons.

1. Many sRNA classes, such as piRNA, tRNA, and rRNA-derived fragments (tRFs/rRFs), are relatively ill defined (Nätt and Öst 2020). Therefore, related sRNA sequences are often found across multiple databases commonly used in feature classification.
2. Since many sRNAs originate from repeats and are very short by nature it is impossible to confidently align all to single loci.
3. Some sRNAs are defined by size. Thus, counting overlapping sequences of differing lengths as one feature appears arbitrary.
4. Many sRNA, like tRFs, rRFs, and some miRNA, are post-transcriptionally modified, both at the nucleotide sequence, but also with the addition of chemical groups that can affect library preparation. This may introduce hidden artificial mismatches not recognized by alignment-based methods (Ebhardt et al. 2009; Xu et al. 2019; Kugelberg et al. 2021).

Previous efforts (Supplementary Table S2) to solve some of the problems in sRNA-seq analysis have often focused on providing modular tools and databases to enhance and simplify the analysis often by prioritizing one class over another (Gebert et al. 2017; Wu et al. 2017; Lu et al. 2018; Shi et al. 2018; Aparicio-Puerta et al. 2019; Li et al. 2020).

To illustrate some of the pitfalls in sRNA analyses that can be revealed and resolved using Seqpac, we reanalyzed three published datasets (Fig. 2B–E; Supplementary Figs S3 and S5). It must be emphasized that the problems raised here are not unique for the illustrated studies, but rather demonstrate examples of the widespread problems with feature-based counting strategies.

Kang et al. (2018) (SRA: PRJNA485638) studied samples with known ratios of sRNA from two different species. Re-analysis using Seqpac demonstrated the strengths of preserving sequence integrity to quickly investigate the effect of mismatches, feature classification, and choice of target genome (Supplementary Fig. S3). Exactly how vulnerable alignment-based strategies are, were illustrated by showing more than 800% increase in false-positive alignments when allowing 3 mismatches compared to perfect mapping, using a dataset with completely randomized sequences, but having the same sequence length distribution as Kang et al. (Supplementary Fig. S4). Tong et al. (2020) (PRJNA666144) studied sRNA in extracellular vesicles excreted by different cell lines *in vitro* (Supplementary Fig. S5). Using Seqpac, we revealed a severe bias that was hidden when applying feature-based methods. Lastly, Snoek et al. (2019) (PRJNA413777) studied sRNA in vaginal self-samples of cervical cancer patients. Here we demonstrate how Seqpac can enhance clinical interpretations by accounting for the vaginal microbiota that went undetected by the original analysis (Fig. 2B and C) and strongly affected the experiment (Fig. 2D). More precisely, the relationship resembles how to weed density affects crop yields (Fig. 2E), which seems important to account for when building diagnostic models for this disease.

Finally, it should be emphasized, that we do not claim that feature-based counting is wrong *per se*, but that results are more challenging to interpret and less flexible for further analyses. The goal of Seqpac is primarily to provide a flexible environment for a sequenced-based strategy in R, which can easily take advantage of the open-source utilities already available in R, such as packages, software (e.g. Posit), and communities (e.g. Bioconductor). To illustrate the point, we used the Kang et al. dataset again (Supplementary Fig. S3). This time we classified tRFs using Seqpac. MINTMAP currently provides the best tRF classification, stratifying by cleavage products like halves, 3', 5', and i' fragments (Loher et al. 2017). It is, however, only readily available for human studies. Seqpac provides functions that can be adopted for tRF classification in any species (Supplementary Fig. S1). To illustrate the difference, we compared Seqpac with sRNAtoolbox (Aparicio-Puerta et al. 2019) and a featureCounts workflow (Liao et al. 2014). Adopting the Seqpac-strategy resulted in >150 additional subtypes (Supplementary Fig. S6). Importantly, in Seqpac this analysis can be made at any time, immediately after count table creation or on a heavily filtered, pre-analyzed, dataset. By providing the framework, we hope that the Seqpac environment allows the development of new analyses limited only by the creativity of the user.

Supplementary Table S1 contains a short reference card describing most Seqpac functions and their dependencies. Extended methods are available in Supplementary File S2. All code for reproducing the results is available in Supplementary File S3.

## 3 Discussion

Seqpac is primarily designed for sRNA-seq analysis. Thus, it does not currently support paired-end sequencing. Paired-end sequencing is not required for most sRNA applications where the target sequences seldom exceed 75 nt and where detection of adapter sequence guarantees sRNA.

Nonetheless, we see many advantages for using sequence-based counting strategies also in long-RNA-seq analysis, not only for better data lineages. Such strategies would, for example, make it easier to control for genetic variants, feature read coverage (Nätt et al. 2019; Nätt and Öst 2020), or study fusion transcripts (Conesa et al. 2016). Future studies should therefore aim to optimize sequence-based counting strategies for long-RNA-seq.

Seqpac applies Bowtie (Langmead et al. 2009) as its main aligner. Whilst still the most popular aligner for sRNA, it does not support insertion and deletion (indel) mapping well. This emphasizes the importance of preserving sequence integrity, not only for sequences that align to a target genome but also those that fail to align due to, for example, genetic polymorphism. Despite its limitation, Bowtie is reliable with very short sequences, which is likely the reason for its popularity within the sRNA community. We initially implemented Rsubreads (Liao et al. 2019) in Seqpac, which better supports indel mapping. However, for certain read lengths, we consistently experienced failure to correctly pick the best alignment. Thus, future research should combine reliable indel mapping of sRNA data with preserved sequence integrity.

We have identified limitations in concurrent RNA-seq workflows associated with the widespread use of feature-based counting strategies. This problem is often amplified by complicated, modular, data lineages (many tools, many programming languages). Without a strategy to preserve the integrity of read sequences, such workflows may create black boxes in the analysis that increase the risk of misinterpretation and hidden bias. By building a framework for sRNA-seq analysis—intended to preserve sequence integrity—we have demonstrated the panoptic view needed for making the correct interpretations. We believe that similar strategies may aid in resolving the current replication crisis experienced by many fields that depend on transcriptome-scale data analysis.

## Supplementary Material

btad144_Supplementary_Data

## Data Availability

Seqpac is available at Bioconductor (https://bioconductor.org/packages/seqpac). Raw data used in this article are available at SRA: PRJNA485638, PRJNA666144, and PRJNA413777.
