# Correction to: Seqpac: a framework for sRNA-seq analysis in R using sequence-based counts

**DOI:** 10.1093/bioinformatics/btad379

**Published:** 2023-06-21

This is a correction to: Signe Skog and others, Seqpac: a framework for sRNA-seq analysis in R using sequence-based counts, *Bioinformatics*, Volume 39, Issue 4, April 2023, btad144, https://doi.org/10.1093/bioinformatics/btad144

The following changes have been made to the original publication of this article. Mark-up and statements have been added to indicate equal contribution for authors within the authorship list. In the online Supplementary data section, three files were missing from the Supplementary data zip file. These have now been supplied: “Supplementary file 1 - Supplementary Tables and Figures”, “Supplementary file 2 - Full Methods” and “Supplementary file 3 - R_script”.

