# Supplementary material for: Seqpac: a framework for sRNA-seq analysis in R using sequence-based counts
Source: Bioinformatics. 2023 Mar 21;39(4):btad144. doi: 10.1093/bioinformatics/btad144 (PMC10085512; doi:10.1093/bioinformatics/btad144)
Supplement: btad144_Supplementary_Data [file btad144_supplementary_data.zip › Supplementary file 1 - Supplementary Tables and Figure.pdf]

Supplementary Table 1. Quick reference for Seqpac functions

Family	Description	Function name	Dependencies	Function description
**PAC generation**	Prepares and builds a PAC object from fastq files.	*merge_lanes*	*ShortReads, digest, foreach*	Quickly merges flow-cell lanes from multiple fastq
		make_counts	*foreach, ShortReads*	Reads fastq, calls *make_trim* or *make_cutadapt,* performs low-level filter, and counts sequences.
		make_trim	*foreach, Biostrings*	Adaptor trimming and fastq quality filter using R internal packages
		make_cutadapt	*foreach, cutadapt, fastq_quality_filter*	Adaptor trimming and fastq quality filter using externally installed software
		make_pheno	-	Prepares user provided phenotype table containing sample information.
		make_PAC	-	Builds PAC object with Pheno, Anno and Counts tables.
		PAC_check	-	Checks if a PAC object is compatible with Seqpac functions
**PAC annotation**	Sequence annotation by aligning against fasta references or overlap with genomic coordinates.	*map_reanno*	*Rbowtie/bowtie*	Annotates PAC sequences through progressive mismatch cycles.
		import_reanno	*foreach, tibble*	Called by *map_reanno* to import bowtie output into R.
		make_reanno	*foreach, tibble*	Builds a reannotation object with genome coordinates and/or fasta sequence names.
		add_reanno	-	Adds mapping coordinates and/or unordered classifications to a PAC object.
		simplify_reanno	-	Makes hierarchical classification of unordered classes obtained from *add_reanno.*
		PAC_gtf	*tibble, rtracklayer, GenomicRanges*	Overlaps genomic coordinates of PAC sequences with features of a gtf/gff file.
		PAC_mapper		Backdoor to the reanno workflow for fast mapping of PAC sequences to obtain a map object.
		map_rangetype		Classifies sequences in the map object according to ranges or secondary structures (e.g. 5', i', 3')
**PAC analysis** *(preprocessing)*	Filtering and normalization.	*PAC_filter*	-	Subsets data by targeting objects or coverage thresholds.
		PAC_filtersep	-	Extracts sequences reaching a threshold within groups of a pheno_target object.
		PAC_norm	*DESeq2*	Normalize a raw counts table and saves it in PAC norm.
**PAC analysis** *(statistics)*	Performs statistical analyses and visualizations.	*PAC_summary*	-	Simple summaries using pheno_targets (means, sd, se, %diff, log2fc) saved in PAC summary.
		PAC_deseq	*foreach, DESeq2*	Prepares, performs and plots DESeq2 analysis from PAC object.
		PAC_pca	*FactoMineR, factoextra*	Performs principal component analysis and plots the results.
		PAC_saturation	*foreach, ggplot2*	Performs a sequence saturation analysis and plots the results.
**PAC analysis** *(visualization)*	Generates graphs and saves processed data summarized over both phenotype and annotations.	*PAC_pie*	*ggplot2, cowplot; grDevices*	Pie-plots using pheno_target and anno_target objects.
		PAC_stackbar	*ggplot2, reshape2, grDevices*	Stacked bar diagrams using pheno_target and anno_target objects.
		PAC_jitter	*ggplot2*	Jitter plots using pheno_target and anno_target objects.
		PAC_nbias	*ggplot2, grDevices*	Size distributed histogram stacked by nucleotide at a defined position (e.g. 1st nucleotide bias).
		PAC_sizedist	*ggplot2, grDevices*	Size distributed bars stacked by an anno_target column (e.g. miRNA/piRNA size distributions).
		PAC_covplot	*ggplot2, reshape2, grDevices, GenomicRanges*	Plots PAC sequence coverage over a reference sequence such as a tRNA or rRNA.
		PAC_trna	*ggplot2, reshape2, grDevices*	tRNA analysis using range-classes obtained from *PAC_mapper/map_rangetype*

All functions are described in detail in the manual for each function (e.g. '?make_counts' in the R terminal) and are exemplified in the Seqpac vignette; 'vignette("seqpac")' in R terminal). Available RRID identification numbers: Biostrings = SCR_016949, Cutadapt = SCR_011841, Rbowtie/bowtie = SCR_005476, tibble = SCR_019186, GenomicRanges = SCR_000025, DESeq2 = SCR_015687, FactoMineR = SCR_014602, Factoextra = SCR_016692, ggplot2 = SCR_014601 , cowplot = SCR_018081, GenomicRanges = SCR_000025.

(*1-16*)

Supplementary Table 2. Comparision beween Seqpac and similar tools

	Seqpac	sRNAbench (sRNA toolbox)	miRGe	SPORTS
**Citation**	This manuscript	Barturen, et al. (2014), Aparicio-Puerta, et al. (2019)	Lu, et al. (2018), Looney, et al.(2021)	Shi, et al. (2018)
**Interface**	R	Web based, command line	Command line	Command line
**sRNA class focus**	All sRNA, tsRNA	miRNA	miRNA/tsRNA	All sRNA, tsRNA, rsRNA
**Counting unique sequences?**	Yes	Yes	Yes	Yes
**Annotation strategy**	Flexible hierarchy	Hierarchy	Hierarchy	Hierarchy
**Saves multiple mappings?**	Yes	Yes – if defined	Yes	Yes
**Read sequences accessible?**	Yes	Yes	Yes	Yes
**Visualization**	Plot functions (e.g. PAC_covplot)	Autogenerated png graphical output	Autogenerated PDF graphical output	Autogenerated PDF graphical output
**Output**	PAC object (summary reports, results tables)	Separate reports, png images	Reporting files, PDF images	Separate reports, PDF images
**Special features**	Flexible analysis of sample meta-data and specific annotation features	Extensive profiling and analysis of miRNA	Extensive profiling and analysis of miRNA and tRNA	Simultaneous analysis of all user-defined sRNAs, mismatch analysis

More information: sRNAtoolbox: (*17*), miRGe: (*18*),SPORTS: (*19*)

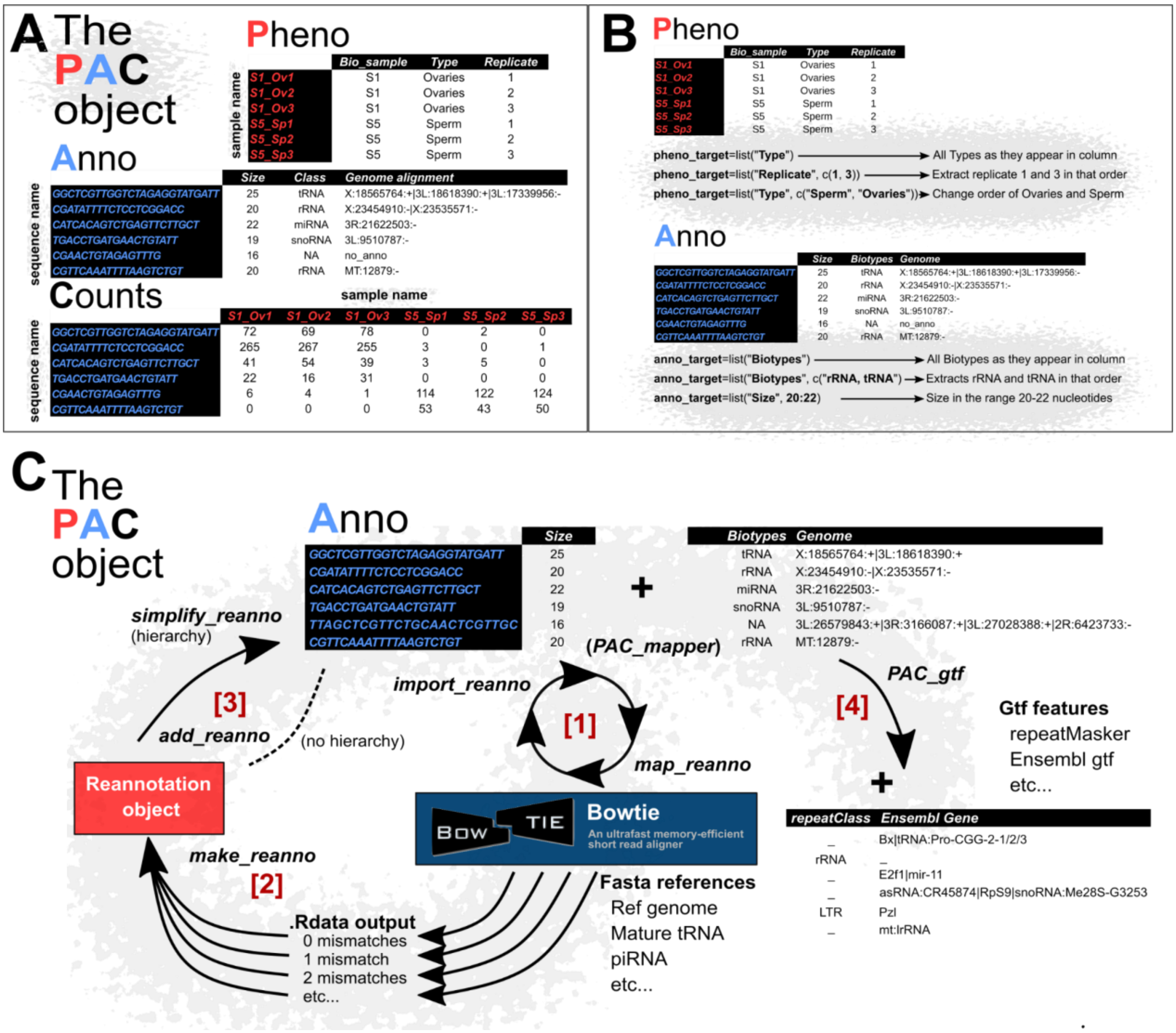

Supplementary Figure 1. Seqpac objects and procedures. Seqpac is designed to preserve the integrity of the raw sequence in the analysis of RNA-seq data. **(A)** Shows a PAC object. In its simplest form PAC contains three tables: the *Pheno* table with sample information; the *Anno* table with sequence information, and the *Counts* table containing the counts of sequences across samples. At more advanced stages a PAC may hold additional tables, like normalized and summarized (e.g. means of treatment groups) tables. **(B)** Targeting objects. Many Seqpac functions applies a novel system for grouping and sub-dividing samples (*Pheno*) and sequences (*Anno*) in a PAC object. This system relies on small targeting objects, which targets information either in the *Pheno* (*pheno_target*) or *Anno* (*anno_target*) tables. A targeting object is a list with two-character inputs. The first points out a column in the target table, and the second points to the entries of that column. The second input is order sensitive. Thus, users can easily rearrange outputs, like the order of groups and sequence classes in a graph. **(C)** For annotating read sequences, Seqpac provides the re-annotation workflow. The *map_reanno* function uses *Bowtie* to align PAC sequences against references sequences, e.g. species genome or RNA database (fasta references) [1]. To promote the mapping efficiency without compromising the user-experience the re-annotation workflow runs in cycles. Each cycle introduces 1 additional mismatch, where only sequences with no alignment proceed to the next cycle. This enables progressive status updates during lengthy alignments and efficient handle of sequences from multiple

references and samples. A reanno object [2] is then generated using *make_reanno*, from which new annotations can be added to the PAC annotation table using the *add_reanno* function. The resulting annotations, which may contain multiple annotations for the same sequence, may be simplified hierarchically into one-annotation-per-sequence using the *simplify_reanno* function [3]. The *PAC_mapper* function is a convenient wrapper for smaller reference sequences (e.g. tRNAs or rRNAs) that will automatically generate *Bowtie* indexes. After a PAC object has been aligned to a genome, the *PAC_gtf* can be used to overlap genomic coordinates with known coordinates of genomic features, e.g. repeats and protein coding genes [4] simulating a feature-based strategy. More details are available in Seqpac's vignette. Example scripts are also available in Supp. file 3.

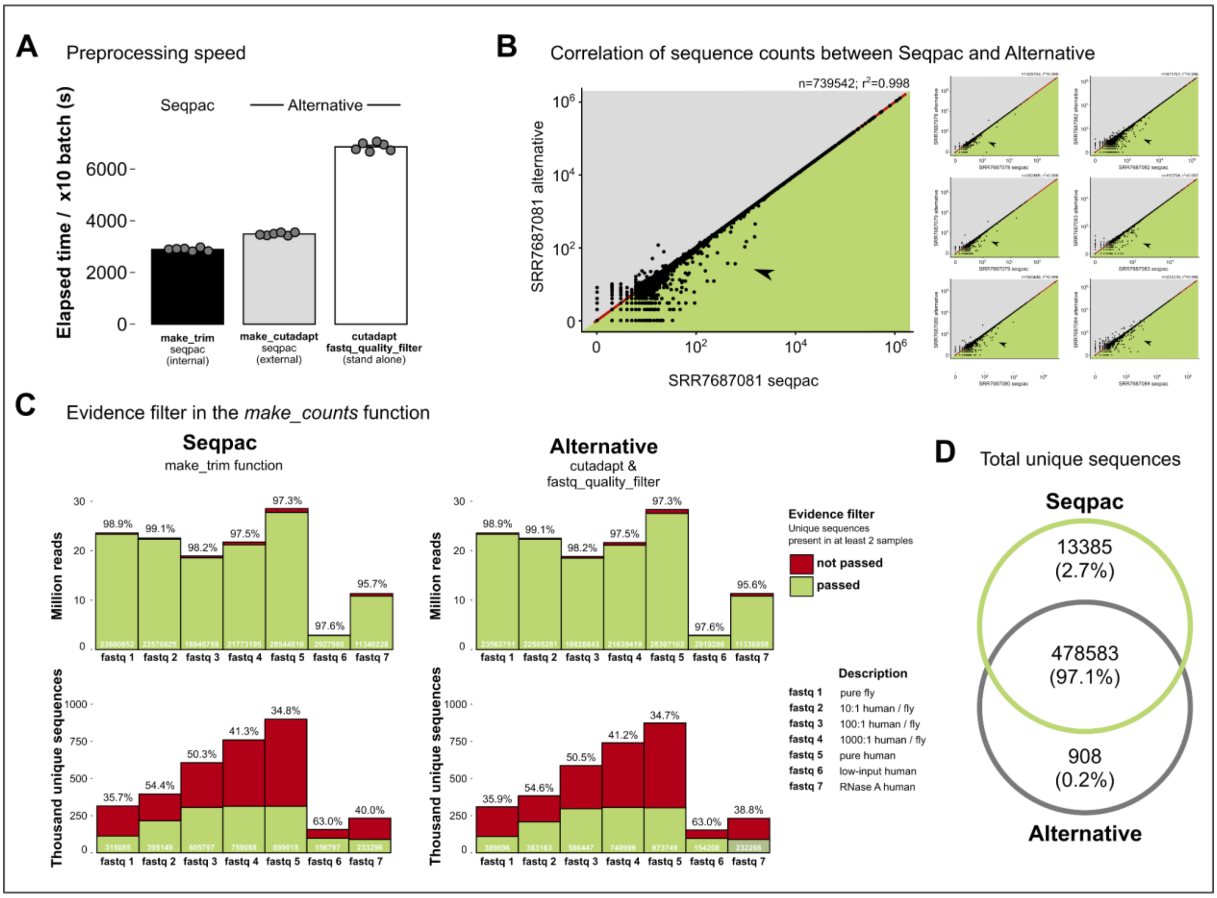

Supplementary Figure 2. Benchmark Seqpac trimming. Performance of Seqpac's internal trimming and counting functions. Seqpac contains multiple options for trimming and filtering prior to generating a count table. The *make_counts* function counts sequences of already trimmed fastq files or calls the *make_trim* function for adaptor trimming prior to counting. Using the Kang *et al.* 2018 dataset (SRA access: PRJNA485638), **(A)** shows the side-by-side processing time for Seqpac's trimming functions and a popular alternative based on the *cutadapt* and *fastq_quality_filter* tools. The test involved 7 fastq files iterated 10 times over 6 batches per function using 7 parallel processes. **(B-D)** Further evaluation of the performance of *make_trim* in terms of the output dataset. **(B)** While sequence counts strongly correlated with the alternative trimming and filtering workflow, *make_trim* generated higher counts (arrows) more often. **(C)** The *make_counts* function contains an evidence filter, which in default mode discards sequences that fail to replicate in at least two independent fastq files. Normally, this low-level filter maintains most reads (top bars), while limiting the sequence diversity (bottom bars). Venn-diagram **(D)** showing a slightly higher ratio of replicable sequences unique to the *make_trim* workflow compared to the cutadapt alternative. Careful inspection of these sequences revealed an inability of cutadapt to identify concatemer adaptor sequences. Concatemer (chimeric) adaptors are found in small quantities in most experiments and are technical constructs, where an incomplete adaptor associates with a complete adaptor during synthesis (*20*). Thus, Seqpac's internal trimming performed better than the popular cutadapt alternative. Scripts for replicating the analyses are available in Supp. file 3.

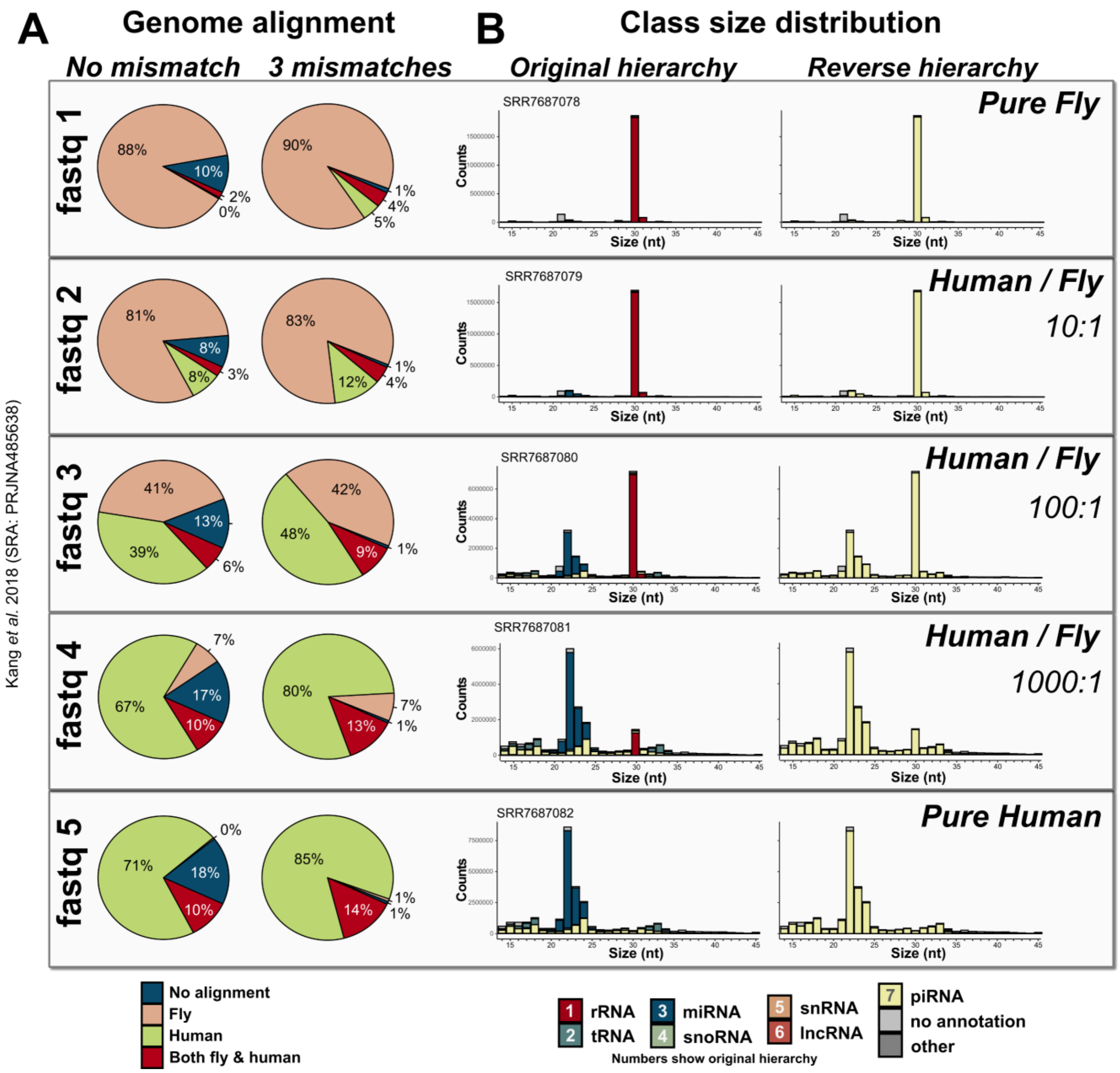

Supplementary Figure 3. Testing the effect of mismatches using Seqpac. Kang *et al.* 2018 (SRA access: PRJNA485638) studied samples with known ratios of sRNA from two species, human and fruit fly. **(A)** Using Seqpac's reannotation pipeline, that is designed to pick the best alignments across multiple references (here: multiple genomes), ratios were accounted for. Allowing 3 mismatches decreased the percent of sequences that failed to align to any of the species, but also increased the frequency that aligned to both. Supplementary Figure 4 shows the dramatic increase in false-positive alignments when allowing up to 3 mismatches. **(B)** Size distributions revealed that fruit fly samples were enriched with a 30 nt fragment. Since Seqpac preserves sequence integrity, sequences was BLASTed only to find that they came from the 2S rRNA subunit. To avoid redundant sequencing, 2S is often removed during library preparation in this species. When prioritizing miRNA over piRNA, samples containing human RNA were enriched with correctly sized miRNA. Reversing the hierarchy turned nearly all sequences into piRNA. All code for reproducing the analysis is available in Supp. file 3

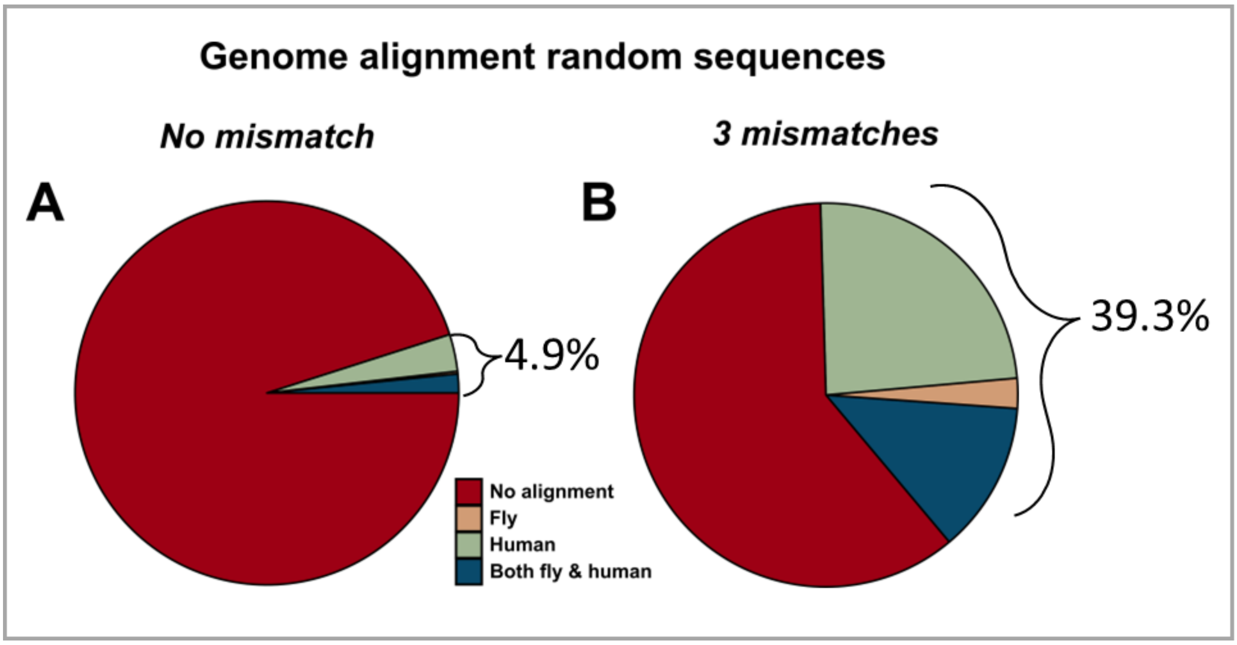

Supplementary Figure 4. Assessment of false-positive alignments in sRNA data. Using the Kang *et al.* 2018 samples presented in Supp. Fig. 2 and 3 (SRA access: PRJNA485638) as starting point, we generated a dataset with random sequences. This was repeated 20 times, resulting in 100 samples (5x20) with random sequences of the same length and same count distributions as the original dataset. This dataset was then aligned against the fly (dm6) and human (hg38) genomes as in Fig. 2A allowing for 0 **(A)** and 3 mismatches **(B)**. Even without mismatches, the dataset resulted in substantial false positive alignments in both species but to a greater extent for the larger human genome **(A).** Allowing for 3 mismatches increased false-positive alignments with >800% **(B).** All code for reproducing the analysis is available in Supp. file 3.

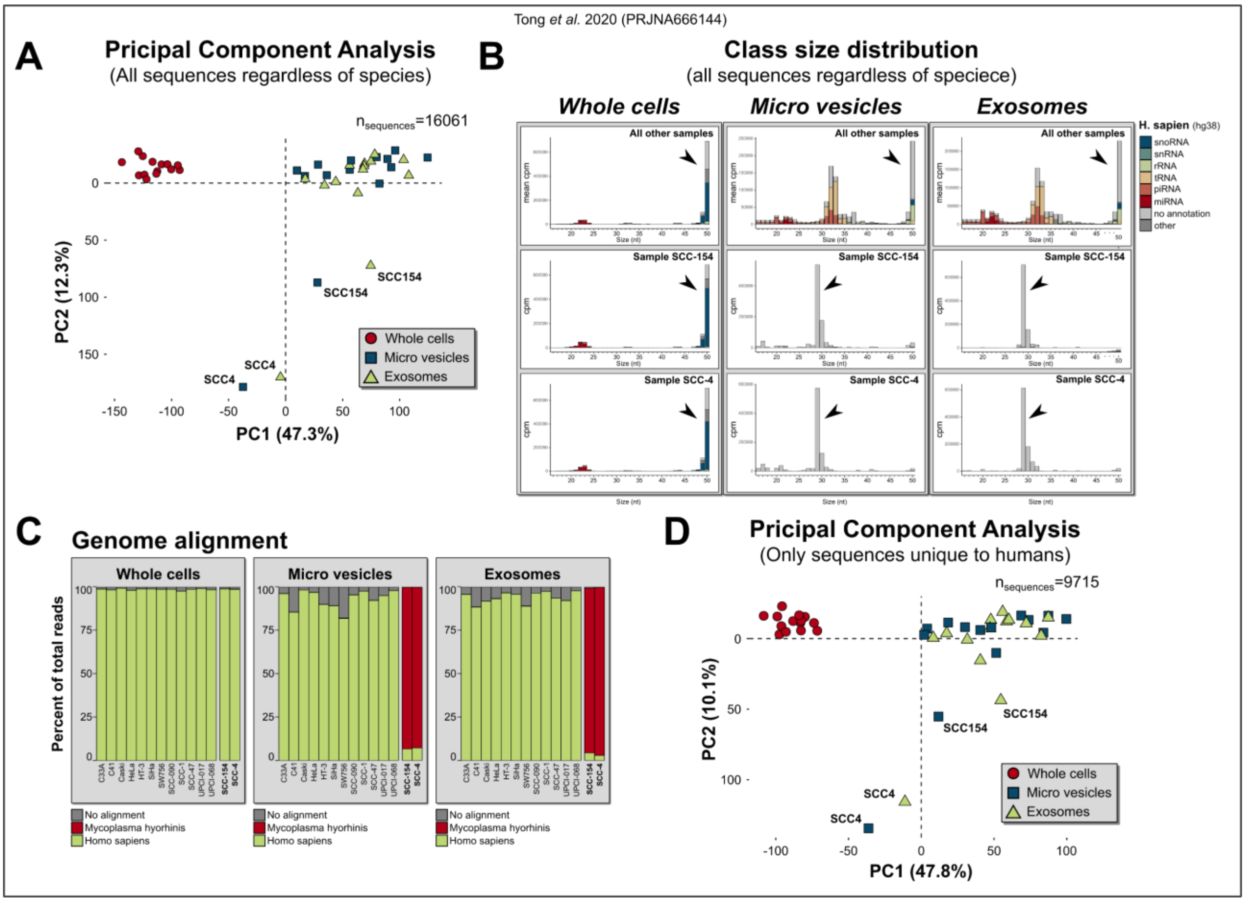

Supplementary Figure 5. Seqpac identifies critical factors hidden using a feature-based strategy. Tong *et al.* 2020 (PRJNA6661144) studied extra-cellular vesicles (micro vesicles=MV and exosomes=EXO) excreted by different cancer cell lines using a feature-based strategy. Among other things, they concluded that "snoRNA was most abundant in cells, while only representing a small fraction" in MV's and EXO's. The authors do not report of any contamination but disclaim: "our study is limited by the purity of isolated EXOs and MVs". **(A)** A Principal component analysis (PCA) of all sequences appearing with at least 10 counts across 50% of the samples (regardless of species) identified extracellular vesicles from SCC4 and SCC154 cells as outliers. **(B)** Size distribution revealed that most reads were >=50 nucleotides. Four vesicle samples from two cell lines, SCC4 and SCC154, mostly failed to align against known human sRNA. **(C)** Multi-genome alignment revealed severe contamination by *Mycoplasma* in these samples. **(D)** An almost identical scatter plot as in (A) was observed when PCA was conducted on re-normalized data after removing all *Mycoplasma* sequences and only proceeding with sequences aligning with the human genome. This mimics the normal procedure in feature-based strategies and indicates that bias may remain despite removal of all non-human sequences. All PCA was generated using variance stabilized transformations (vst). The following Seqpac functions were applied: PAC_filter, PAC_norm, PAC_pca, PAC_sizedist, PAC_stackbar. Details of how to perform the analysis from untrimmed fastq files are available in Supp. File 3

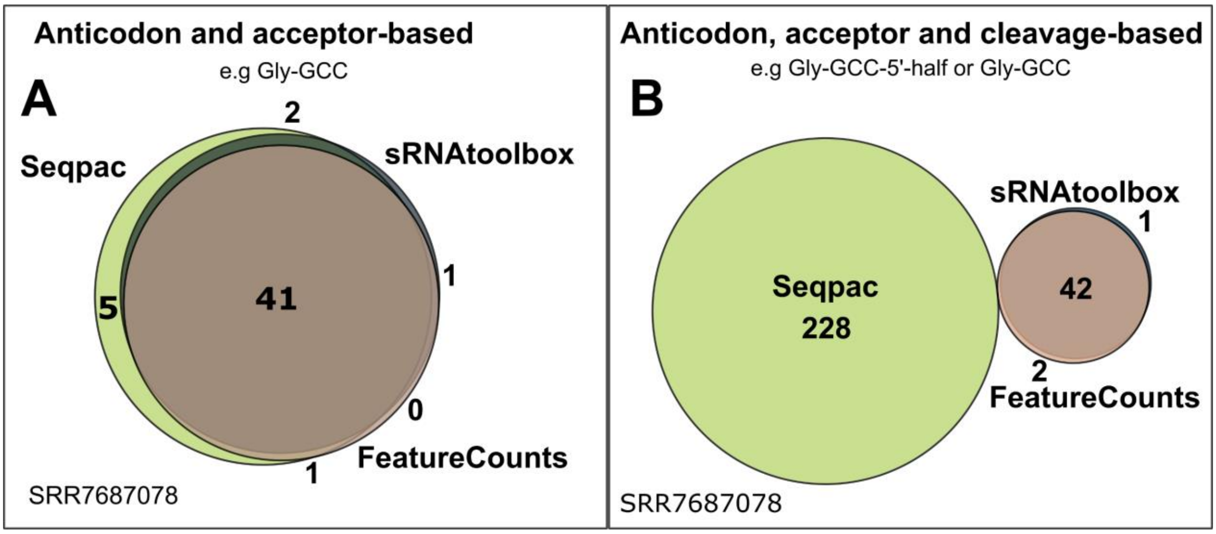

Supplementary Figure 6. Seqpac identifies more unique tRFs compared to other sRNA strategies.

To demonstrate the flexibility in using Seqpac's sequence-based environment we built near-as-possible identical workflows in Seqpac, sRNAbench (sRNA toolbox; (*17*)) and FeatureCounts (*21*) and loaded one of the samples (SRR7687078) from the Kang *et. al.* dataset (Supp. Fig. 3). For each workflow we tried to classify tRNA isodecoder and acceptor, as well as cleavage fragments (tRFs). **(A)** Workflows performed similarly in identifying decoders and acceptors. **(B)** Seqpac found more unique tRFs and distinguishes the fragments in more details. The other stops on the 42 isoacceptor/decoder classifications. This exemplifies the flexibility in using the Seqpac environment for advanced classification, based on secondary structure. This workflow applies the PAC_mapper, map_rangetype, function bundle (Supp. Tab. 1). For an overview on more differences between Seqpac and other tools please see Supp. Tab. 2. All code for reproducing the analysis is available in Supp. File 3.

References

1. R. Rivest, S. Dusse. (MIT Laboratory for Computer Science Cambridge, 1992).
2. H. Ooi, S. Weston, Microsoft. (2020), vol. R package version 1.5.0.
3. H. Pages, P. Aboyoun, R. Gentleman, S. DebRoy, Biostrings: String objects representing biological sequences, and matching algorithms v2.48.0. *R package*, (2018).
4. M. Martin, Cutadapt removes adapter sequences from high-throughput sequencing reads. *2011* **17**, 3 (2011).
5. G. Hannon, A. Gordon, etc. (2010).
6. F. Hahne, A. Lerch, M. Stadler. (2012).
7. H. Wickham *et al.*, Welcome to the Tidyverse. *Journal of Open Source Software* **4**, 1686 (2019).
8. M. Lawrence *et al.*, Software for computing and annotating genomic ranges. *PLoS computational biology* **9**, e1003118 (2013).
9. M. I. Love, W. Huber, S. Anders, Moderated estimation of fold change and dispersion for RNA-seq data with DESeq2. *Genome Biology* **15**, 550 (2014).
10. M. Lawrence, R. Gentleman, V. Carey, rtracklayer: an R package for interfacing with genome browsers. *Bioinformatics* **25**, 1841-1842 (2009).
11. S. Lê, J. Josse, F. Husson, FactoMineR: an R package for multivariate analysis. *Journal of statistical software* **25**, 1-18 (2008).
12. A. Kassambara, F. Mundt, Package 'factoextra'. *Extract and visualize the results of multivariate data analyses* **76**, (2017).
13. H. Wickham, *ggplot2: elegant graphics for data analysis*. (Springer, 2016).
14. C. O. Wilke, cowplot: streamlined plot theme and plot annotations for 'ggplot2'. *R package version 0.9* **4**, (2019).
15. R. C. Team, R: A language and environment for statistical computing. (2013).
16. H. Wickham, Reshaping data with the reshape package. *Journal of statistical software* **21**, 1-20 (2007).
17. E. Aparicio-Puerta *et al.*, sRNAbench and sRNAtoolbox 2019: intuitive fast small RNA profiling and differential expression. *Nucleic Acids Research* **47**, W530-W535 (2019).
18. Y. Lu, A. S. Baras, M. K. Halushka, miRge 2.0 for comprehensive analysis of microRNA sequencing data. *BMC Bioinformatics* **19**, 275 (2018).
19. J. Shi, E.-A. Ko, K. M. Sanders, Q. Chen, T. Zhou, SPORTS1. 0: a tool for annotating and profiling non-coding RNAs optimized for rRNA-and tRNA-derived small RNAs. *Genomics, proteomics & bioinformatics* **16**, 144-151 (2018).
20. M. Kircher, P. Heyn, J. Kelso, Addressing challenges in the production and analysis of illumina sequencing data. *BMC genomics* **12**, 1-14 (2011).
21. Y. Liao, G. K. Smyth, W. Shi, featureCounts: an efficient general purpose program for assigning sequence reads to genomic features. *Bioinformatics* **30**, 923-930 (2014).
