# Supplementary material for: Seqpac: a framework for sRNA-seq analysis in R using sequence-based counts
Source: Bioinformatics. 2023 Mar 21;39(4):btad144. doi: 10.1093/bioinformatics/btad144 (PMC10085512; doi:10.1093/bioinformatics/btad144)
Supplement: btad144_Supplementary_Data [file btad144_supplementary_data.zip › Supplementary file 2 - Full methods2.pdf]

Supplementary file 2 – Full Methods

1. Package development

Instructions on how to download and install Seqpac is available at Bioconductor (https://bioconductor.org/packages/seqpac). Dependencies for the main Seqpac functions are listed in Supp. tab. 1. Seqpac was originally developed and tested on a Linux Mint v.19.1 computer using R 3.4.4 in RStudio 1.2.1335 and devtools 2.3.2. The computer had an Intel Core i7-9800X CPU at 3.8 GHz (8 cores with in total 16 threads) and contained 94 Gb of ram memory. All R internal functions (the analysis in Supp. file 3) were subsequently tested on multiple Windows 10 computers with varying hardware setup using R 3.6.3, 4.0.1 and 4.1.2.

2. Testing Seqpac using published datasets

Fastq files for datasets were accessed through Sequence Reads Archive (SRA) and European Reads Archive (ENA). All code for processing and generating the results are available in Supp. file 3. A brief explanation is provided below.

2.1.1 Kang *et al.* 2018 – Benchmark the trimming (Supp. Fig. 2)

Kang *et al.* (Kang, et al., 2018) (SRA accession: PRJNA485638; ENA download: https://www.ebi.ac.uk/ena/browser/view/PRJNA485638) was used for benchmarking Seqpac's *make_trim* function against two similar workflows (Supp. file 1). In both alternative workflows, system calls to *cutadapt* (Martin, 2011) and *fastq_quality_filter* (in FASTX-Toolkit; http://hannonlab.cshl.edu/fastx_toolkit/) (Hannon, et al., 2010) were made from within R. The first used the *make_cutadapt* function to replicate the parallelization for *make_trim* using the *foreach* package (Ooi, et al., 2020), while the second used the internal parallelization option in *cutadapt*. System time was monitored over 10 iterations replicated 6 times using the *rbenchmark* package (Kusnierczyk, 2012).

PAC objects with counts from trimming/filtering using the *make_trim* function and cutadapt/fasq_quality_filter alternative, were generated using the *make_counts* function either with *trimming="seqpac"* or *trimming="cutadapt"*. To assure that only sRNA was include, since this dataset was generated from a 75 cycle flow-cell, we removed reads that failed to contain adaptor sequence and only kept reads <=45 nt.

The counts table was then imported into a standard PAC object (using the *make_counts* > *make_pheno* > *make_PAC functions*). As phenotypic input file for *make_pheno* function we used metadata downloaded from SRA/ENA (Supp. file 3).

2.1.2 Kang *et al.* 2018 – Multigenome mismatch and length distribution (Supp. Fig. 3)

After benchmarking the trimming algorithm, only the PAC object produced by Seqpac's internal trimming function (make_trim) was used in sequence classification using Seqpac's re-annotation workflow (Supp. Fig. 1). The low-input and degraded samples were excluded from the analysis. The re-annotation workflow uses four functions to align (map_reanno), import (make_reanno), classify (add_reanno) and simplify (simplify_reanno). For more information and examples see Supp. file 3, as well as the vignette guide (available at Bioconductor). Re-annotation against either the human and fly reference genomes or sRNA class references were applied. For genome alignment we downloaded *Homo sapiens* GRCh38.v106 (hg38) and *Drosophila melanogaster* BDGP6.32 (dm6) as fasta references at Ensembl ftp (Yates, et al., 2019)(http://www.ensembl.org/info/data/ftp/).

For the sRNA class alignment, we downloaded fasta references from miRbase v21 (miRNA) (Kozomara, et al., 2018), pirBase.v3 (piRNA) (Wang, et al., 2018), GtRNAdb v18 (tRNA) (Chan and Lowe, 2015) and Ensembl human /fly v106/32 (many types of ncRNA) (Yates, et al., 2019) databases. After generating a reanno objects we used the *simplify_reanno* function to apply different hierarchies in the classification.

To estimate the false-positive contribution of the genome alignments, we used the Kang *et al.* dataset to construct a dataset with completely randomized sequences. As in the previous analysis the low-input and degraded samples were excluded. The sequences in the PAC objects were randomized with resample and the randomized PAC were annotated against the human and fruit fly genomes using Seqpac's reannotation workflow as described above. This was repeated 20 times resulting in a dataset with 100 randomized samples of the same length and count distribution as presented in Supp. Fig 3. Without the degraded and low-input samples, the original

data contained 5 fastq files, resulting in 100 randomized iterations(20x5=100). All code for reproducing the analysis is available in Supp. file 3.

2.1.2 Kang *et al.* 2018 – Comparing Seqpac with two other sRNA-tools (Supp. Fig. 6)

To compare how Seqpac and two other popular sRNA analysis strategies perform on tRNA analysis, we used the SRR7687078 fastq file from Kang *et al.* (Kang, et al., 2018). This fastq file was trimmed as described above using Seqpac's make_trim function. To harmonize each analysis with the others, the workflows were conducted using the following settings:

1. *Seqpac*: Sequences were counted using make_counts with default phred +33 with a score of 20. Map_reanno: mismatch 1, parse_internal="a=TRUE, f=TRUE". PAC_filter: threshold = 100 coverage=2, size = c(15,75). The sample was aligned references for *Drosophila* ncRNA (BDGP6.ncrna and mirbase_22.1). For the basic analysis (Supp. Fig. 6A), a tRNA reference was obtained from RNAcentral (https://rnacentral.org/), for protein-coding (pc) genes BDGP6.32.cdna was used. A hierarchy was applied with simplify_reanno: miRNA, hairpin, mature, rRNA, tRNA, lncRNA, snoRNA, snRNA, sprRNA, piRNA, rnase, pc, scaRNA, asRNA, ncRNA, other RNA. For advanced tRNA analysis (Supp. Fig 6B), the default tRNA workflow was performed against a reference taken from gtRNADB v.19 (Chan and Lowe, 2015).
2. *sRNAtoolbox* (*sRNAbench*): The web browser interface was used. Mapping of trimmed reads was done against similar references as described above. Quality control was phred +33 with a score of 20 using the default parameters, such as a read filter of 2. From this, the sRNA biotype and counts were extracted for each sequence from the "reads.annotation" file from the generated output. The biotype information was collapsed under an identical hierarchy as used in the Seqpac workflow.
3. *FeatureCounts*: FeatureCounts within the Galaxy web-based server was used to map reads against BDGP6.32 with default options. The output was imported into R, where the annotation and count information were extracted. Genes with less than two reads were discarded. From this, tRNA annotation was extracted.

The tRNA annotation of the three workflows were then compared and overlaps were visualized with DeepVenn (Hulsen, 2022).

2.2 Tong *et al.* 2020 – Hidden contamination (Fig 2C-E)

Tong *et al.* (Tong, et al., 2020) (SRA accession: PRJNA666144; ENA download: https://www.ebi.ac.uk/ena/browser/view/PRJNA666144) was used to demonstrate some pitfalls with feature-based counting. PAC generation and re-annotation was performed similarly to the Kang *et al.* dataset (section 2.1) with a few exceptions. Since the Tong *et al.* dataset was generated from a 50 cycle flow-cell we did not remove reads that failed to contain adapter sequence and did not filter by max read length. Longer reads allow for inter-adapter length validation, where detecting the adapter sequence in the read guarantees that it originated from short RNA. To investigate if long RNA may have biased the results in Tong *et al.* we included all read lengths in the analysis. Analysis and graphs were generated using the *PAC_pca*, *PAC_sizedist* and *PAC_stackbar* functions. Initial alignment was done using Human GRCh38.v106 (hg38). After the initial analysis, we BLASTed highly expressed sequences that failed to align to the human genome at NCBI (Sayers, et al., 2022). This gave perfect mapping to the *Mycoplasma hyorhinis* ATCC (ASM38351v1) genome. Then, we downloaded and aligned this genome besides the human using Seqpac's re-annotation workflow. This picks the best hit, with the fewest mismatches, from either genome.

2.3 Snoek *et al.* 2019 – Exploring the microbiota (Fig 2B-E)

Snoek *et al.* (Snoek, et al., 2019) (SRA accession: SRP119662; ENA download https://www.ebi.ac.uk/ena/browser/view/PRJNA413777). This is the largest publicly available sRNA dataset from cervical cancer patients. We used this dataset to explore the hidden contribution of the microbiota in clinical self-samples using Seqpac. To trim and make a PAC object from this extensive and variable dataset (fastq sizes from 0.51 to 4.47 Gb) using a Windows laptop computer (Intel(R) Core(TM) i7-8650U CPU at 1.90GHz, 4 cores/8 threads, with 16.0 GB RAM) we applied the *chunk_size* and *on_disk* options in *make_trim*/*make_counts* functions (Supp. file 3). Using these option Seqpac may process trimming and counting in chunks, saving the results temporally on disk instead of in memory. These options can be applied when processing challenging data, but on the expense of time. We,

therefore, strongly recommend over-night processing. In addition, we applied adapter trimming and counting separately, saving the trimmed fastq files (*make_trim), before* counting these files at a different timepoint (*make_counts*). After initial alignment to the human genome, we BLASTed sequences with no alignment at NCBI (Sayers, et al., 2022). Nearly all aligned with species in the vaginal microbiota, of which most came from the Lactobacillus genus. We downloaded the genomes of some of these species.

Lactobacillus: *acidophilus* (GCF_000389675.2_ASM38967v2), *crispatus* (GCF_009769205.1_ASM976920v1), *gasseri* (GCF_008868535.1_ASM886853v1), *iners* (GCF_009556455.1_ASM955645v1).
Candida: *albicans* (GCF_000182965.3), *glabrata* (GCF_000002545.3_ASM254v2), *tropicalis* (GCF_000006335.3_ASM633v3).
Bifidobacterium *longum* (GCF_000196555.1_ASM19655v1),
Dialister *hominis* (GCF_007164725.1_ASM716472v1),
Prevotella *buccalis* (GCF_000177075.1_ASM17707v1),
Megasphaera *hexanoica* (GCF_012843505.1_ASM1284350v1),
Gardnerella *vaginalis* (GCF_002861965.1_ASM286196v1) and aligned them in parallel with human genome (GRCh38) through Seqpac's re-annotation workflow (Supp. Fig 1C). Then we applied the PAC_stackbar and PAC_pca functions (Supp. Tab 1). To explore the underlying contribution of the microbiota to experimental-wide variance, we extracted the scores of the first principal component from each patient and plotted these against the percent microbiota detected. After visual inspection, we decided to apply a non-parametric yield-loss regression model using the aomisc/drc packages (Onofri, 2020; Ritz, et al., 2015) and then tested the goodness-of-fit of the model.

For all studies, as phenotypic input file for the make_pheno function we used metadata downloaded from SRA/ENA (Supp. file 3).

References

Chan, P.P. and Lowe, T.M. GtRNAdb 2.0: an expanded database of transfer RNA genes identified in complete and draft genomes. *Nucleic Acids Research* 2015;44(D1):D184-D189.
Hannon, G., Gordon, A. and etc. 2010. FASTX-Toolkit - FASTQ/A short-reads pre-processing tools. Release 0.0.13. http://hannonlab.cshl.edu/fastx_toolkit/

Hulsen, T. DeepVenn--a web application for the creation of area-proportional Venn diagrams using the deep learning framework Tensorflow. js. *arXiv preprint arXiv:2210.04597* 2022.
Kang, W.*, et al.* miRTrace reveals the organismal origins of microRNA sequencing data. *Genome biology* 2018;19(1):1-15.
Kozomara, A., Birgaoanu, M. and Griffiths-Jones, S. miRBase: from microRNA sequences to function. *Nucleic Acids Research* 2018;47(D1):D155-D162.
Kusnierczyk, W. 2012. rbenchmark: Benchmarking routine for R. Release 1.0.0.

. https://CRAN.R-project.org/package=rbenchmark
Martin, M. Cutadapt removes adapter sequences from high-throughput sequencing reads. *2011* 2011;17(1):3.
Onofri. The broken bridge between biologists and statisticians: a blog and R package. In.: Statforbiology, IT; 2020.
Ooi, H., Weston, S. and Microsoft. foreach: Provides Foreach Looping Construct. In.; 2020.
Ritz, C.*, et al.* Dose-response analysis using R. *PloS one* 2015;10(12):e0146021.
Sayers, E.W.*, et al.* Database resources of the national center for biotechnology information. *Nucleic Acids Res* 2022;50(D1):D20-d26.
Snoek, B.C.*, et al.* Genome-wide microRNA analysis of HPV-positive self-samples yields novel triage markers for early detection of cervical cancer. *Int J Cancer* 2019;144(2):372-379.
Tong, F.*, et al.* Comprehensive profiling of extracellular RNA in HPV-induced cancers using an improved pipeline for small RNA-seq analysis. *Sci Rep* 2020;10(1):19450.
Wang, J.*, et al.* piRBase: a comprehensive database of piRNA sequences. *Nucleic Acids Research* 2018;47(D1):D175-D180.
Yates, A.D.*, et al.* Ensembl 2020. *Nucleic Acids Research* 2019;48(D1):D682-D688.
